# Material durability, material failure, and material investment—the complexity of concrete

**DOI:** 10.1038/s44172-024-00172-w

**Published:** 2024-02-03

**Authors:** John L. Provis

**Affiliations:** Paul Scherrer Institut, Laboratory for Waste Management, Forschungsstrasse 111, 5232 Villigen PSI, Switzerland

**Keywords:** Civil engineering, Composites

## Abstract

Recent high-profile concrete material failures, including the collapse of parts of public buildings in the UK, have highlighted the need for a greater understanding of the durability of concrete. Here, John Provis explores the need to recognise the complexity of concrete when planning both the research and application of this key construction material.

## Teaser

Recent high-profile concrete material failures, including the collapse of parts of public buildings in the UK, have highlighted the need for investment in understanding and ensuring the durability of concrete. Concrete is a vastly more complex material than most people appreciate, and this needs to be recognised when planning both research and practical application of this key component of our built environment.

## Durability and material failures

Benjamin Franklin was quoted to say “…*in this world nothing can be said to be certain, except death and taxes*”^1^—and in a materials engineering sense, the concept of “death” could very well be understood as a material or component reaching the end of its useful service life and falling below the required level of one or more engineering properties. The knowledge that an item can wear out—and that more or less every item eventually will—is probably as old as the human usage of tools. Thermodynamicists will of course invoke concepts such as entropy to explain this process, which has the advantage of being correct but is rather less poetic.

Nonetheless, and despite the deeply ingrained understanding that nothing lasts forever, some recent cases of infrastructure material failure will be used in this brief discussion piece to highlight the need for research, investment, and societal discourse to support the safe and effective use of engineering materials.

The durability of construction materials in the public estate has come to particular attention recently in the UK with the discovery that concrete elements made from reinforced autoclaved aerated concrete (RAAC), which were used extensively in constructing buildings including schools, hospitals, courthouses, and other public facilities during the 1950s-1990s, were failing catastrophically in service^2^. It is clear that society will not tolerate the collapse of school or hospital ceilings, where children or patients are placed at risk of injury, and so RAAC-containing buildings have been closed and targeted for repair, replacement, or retrofitting. These closures were imposed in some schools immediately before the scheduled start of the 2023-4 school year in England and thus gained a degree of media attention that is very rarely dedicated to questions around concrete durability.

There was some degree of surprise expressed in the UK media around how this had—apparently overnight—become such a major problem in so many buildings without coming to the attention of engineering professionals and/or asset owners. The answer is, almost inevitably in cases such as this, that warnings were provided by engineers who had conducted analysis of the materials years (or decades) earlier^2^, but that the investment required to mitigate the danger posed by materials that had not yet failed was deemed by the asset owners (public bodies with many competing and challenging financial priorities) to be expensive and not sufficiently urgent to support a case for investment^3,4^. This is far from a UK-specific issue; the collapse of the Morandi Bridge in Genoa, Italy^5^ and the Champlain Towers South condominium collapse in Surfside, Florida, USA^6^ are other well-known and tragic recent disasters, and it is widely documented that civil infrastructure is in urgent need of both detailed condition assessment and extensive repair in other parts of the world^7^. Unfortunately, in most countries, government investment priorities tend to lean toward infrastructure repair (or even maintenance) only immediately after a disaster, even when—as in the case of the UK’s RAAC inventory—the materials or structural elements are already well understood to be reaching the end of their service life.

## Complexity and interdisciplinarity

A key complication in dealing with (i.e., assessing, predicting, or testing) the durability of reinforced concrete is that the material under investigation is probably one of the most chemically and microstructurally complex non-biological materials in modern society. Materials scientists and engineers who work with composite materials conventionally discuss two-component composites, and marvel at the complexity of a three- or four-phase composite. Meanwhile, conventional reinforced concrete could realistically be argued to be a composite of 14 or more phases, the physical and chemical interactions of which are all important in defining engineering properties including strength and durability:

- mild steel;
- coarse aggregate (gravel);
- fine aggregate (sand);
- four distinct cement clinker phases (tricalcium silicate, dicalcium silicate, tricalcium aluminate, tetracalcium aluminoferrite), assuming that the rapidly-reacting sulfate phases in the cement have already been consumed;
- at least four distinct families of cementitious hydrates that result from the reaction of the cement with water (calcium silicate hydrate, calcium hydroxide, ettringite-structured calcium sulfoaluminate hydrates, and hydrocalumite-structured or hydrogarnet-structured calcium aluminate hydrates with multiple anion substitutions);
- almost always one or more supplementary cementitious materials (e.g. limestone, blast furnace slag, coal ash, calcined clays, silica fume) which may themselves be mineralogically or chemically heterogeneous
- pore fluid (a concentrated electrolyte solution with a pH often above 12.5);
- entrained or entrapped air.

There are also likely to be surface-active organic molecules present throughout the concrete, which are added to control flow characteristics during mixing and casting, incorporation (or not) of air bubbles/voids, and also used to enhance the grinding of the cement before it is even incorporated into the concrete. Concrete microstructures are heterogeneous and show important features on every length scale from nanometres to centimetres. As a field of materials science and engineering, concrete is therefore incredibly rich in potential scientific detail and complexity—but it is not seen as particularly glamorous in most academic materials engineering programmes, nor is it the type of “game-changer” material that research funders generally seek to target through major strategic investments. Rather, it is a low-cost commodity material that is broadly assumed (by researchers and society) to be able to do its job reliably, with its intricacies and complexity rarely taken into consideration. Related to this topic, the author of this document has in the past held the title Professor of Cement Materials Science and Engineering, which almost inevitably raised questions in conversations—even with fellow engineering professionals—such as “why would we even need a professor of cement? Don’t we already know everything about it?”

This then leads to a fundamental point that is rarely stated, but which underlies a lot of these issues: when the most complex engineering materials are mainly studied and analysed by researchers whose specialisation is not materials science (e.g. the dominance of civil engineers in concrete materials research), important domain-specific understanding and skills (e.g. microstructural and chemical analysis techniques) will not always be able to be transferred or applied effectively. The converse is also true, and was eloquently described by the eminent civil engineer Adam Neville^8^—materials scientists do not always connect their work effectively to the macro-scale realities of construction engineering, including the many demands that are placed on cement and concrete in service. Interdisciplinarity, and collaborations between those who specialise in phenomena taking place at different length scales, are critical here. However, truly effective interdisciplinary research requires funding at a sufficient scale, targeted through major initiatives, and it is very difficult for budget-constrained funders, often working in service to government departments that are seeking good-news stories or breakthrough announcements, to recognise that work on concrete durability is sufficiently urgent or mission-critical to merit this form of support.

## Concrete failure or steel failure?

The majority of practical issues related to the durability of reinforced (or prestressed) concrete relate to the fact that the aqueous corrosion of mild steel can be very effectively postponed—but not actually prevented—by encasing it in highly alkaline concrete^9^. Thermodynamically, mild steel would actually prefer not to exist; its most stable solid form under more or less any conditions in contact with water or air is oxidised, not metallic^10^. An undamaged concrete can significantly delay oxidation (i.e., corrosion/rusting) by protecting and passivating the steel surface, forming an impermeable oxide film on the steel surface that effectively prevents the further progress of oxidation. Unfortunately, the degradation of the concrete by chloride ingress, carbonation, and/or cracking can lead to the breakdown of this film and the initiation of corrosion—and this process tends to be difficult or expensive to diagnose, stop or reverse, particularly when the steel is embedded within a large or difficult-to-access concrete element.

By corollary, this also provides an answer to the increasingly often asked question, “if the Romans could build such durable concrete structures, why don’t we do what they did?”. Among a large number of contributing factors (including survivorship bias in considering the Roman structures we can observe today), the fact that Roman concrete construction did not include embedded steel is an essential difference between their materials and modern reinforced concretes. Modern engineering and architecture rely very heavily on the enormous improvements in flexural and tensile strength that are granted through the design of reinforced concrete as a composite material, and it is not feasible to revert to Roman methods which did not bring these advantages.

Another complicating factor in this question is that the existing level of understanding of steel corrosion in concrete is incomplete and subject to mismatch between laboratory and field data, and in need of very significant research investment to develop the depth of insight that is needed to fully support modern engineering practice^11^. The probabilistic aspects of corrosion-induced failure are essential—prediction of the service life of a reinforced concrete element can actually be undertaken with some accuracy, at least in a probabilistic sense, via setting a performance basis and a specified percentage risk of failure^12^ as a criterion for defining the end of service life and the need for an intervention to assess, repair, replace or retrofit. However, because the allowable failure percentages are very low for safety-critical engineering elements^11,13^ the majority of interventions will be applied to elements that are still in a safe and functional condition—which then leads to the discussion of “why are we wasting money to fix things that don’t need fixing?”, and motivates the need for greatly improved non-destructive testing protocols to assess which elements do actually need engineering intervention. In the case of the UK’s RAAC issues, the identification of which panels are in need of intervention has been complicated by the limitations in applying standard concrete assessment techniques (e.g. coring, covermeters, and radar-based techniques) to foamed materials and in the presence of thermally insulating layers that may be backed by metallic foils, and in locations that may contain asbestos^4^.

## Concluding comments

It is in many ways obvious—and yet also bears regular repetition—that to be able to truly solve a problem, one must first understand its causes. Unfortunately, a piecemeal approach to investment in research into the durability of concrete and concrete elements (including related skills and infrastructure) in many parts of the world means that there are important fundamental pieces of science that do not yet exist, in support of the engineering that critically needs to be done in service of human society. In many ways, the conclusions reached here mirror those of the 1987 U.S. National Research Council report entitled “*Concrete Durability: A Multibillion Dollar Opportunity”*^14^. Considering that 35 years have passed since the publication of that report and that both the digital revolution and the prominence of climate change in the public eye have so vastly changed how research (and society more broadly) operate, it is striking to see how its core messages remain so relevant. We must obviously design materials that are resilient to climate change—both in service and in their impact on the environment during production—and these efforts must also result in materials that are provably durable in the form of a reinforced concrete composite.

The key message of this Comment is that materials don’t last forever; particularly composite materials with steel in them—and reinforced concrete is a composite with steel in it—and we need better tools (physical and intellectual) to enable us to more appropriately and efficiently solve key materials durability problems before, rather than after, they cause harm to people.

Preventative maintenance on structures and elements that have reached the end of their defined (or designed) service life may be expensive and disruptive—but if it is not done, we get catastrophic failures instead. So, it costs enormously more (and without even mentioning the enormous human cost of some structural failures!) to ignore materials science and engineering than it does to fund it properly, and to intervene when intervention is first needed.
